# Sexual and Reproductive Health during the COVID-19 Pandemic: Results from a Cross-Sectional Online Survey in Germany

**DOI:** 10.3390/ijerph19031428

**Published:** 2022-01-27

**Authors:** Jule Räuchle, Peer Briken, Johanna Schröder, Olena Ivanova

**Affiliations:** 1 Institute for Sex Research, Sexual Medicine and Forensic Psychiatry, University Medical Centre Hamburg-Eppendorf, 20246 Hamburg, Germany; j.raeuchle@uke.de (J.R.); briken@uke.de (P.B.); jo.schroeder@uke.de (J.S.); 2 Department of Psychology, Medical School Hamburg, 20457 Hamburg, Germany; 3 Division of Infectious Diseases and Tropical Medicine, Medical Centre of the University of Munich (LMU), 80802 Munich, Germany; 4 German Center for Infection Research (DZIF), Partner Site Munich, 80802 Munich, Germany

**Keywords:** COVID-19, sexual and reproductive health, sexual behaviour, adults, Germany, online

## Abstract

The accumulated evidence maps the COVID-19 pandemic’s diverse impacts on sexual and reproductive health (SRH); however, the precise changes in sexual behaviours and the underlying causes producing these changes are rarely considered. This study is aimed at assessing the changes in sexual behaviours during the COVID-19 pandemic in Germany, using quantitative methods, and it is also aimed at identifying the underlying reasons, using qualitative methods. It is a part of the broader I-SHARE project, which administered a cross-sectional online survey in 33 countries to describe the effects of the COVID-19 restrictions on different aspects of SRH. In the current study, a total of 611 adults from Germany are included. The findings demonstrate a decline in sexual satisfaction, as well as increases in sexual problems and partnership conflicts. Furthermore, the findings indicate an increase in pornography consumption and masturbation. Psychological stress, due to the pandemic, seemed to be the main reason for the changes in the participants’ sexual behaviours, followed by a decrease in social contacts, and an increase in time resources. Thus, it is important to provide accessible clinical and psychosocial (online) interventions and services in order to maintain good sexual health in times of pandemic.

## 1. Introduction

In early 2020, a novel coronavirus—SARS-CoV-2—was identified, which, in addition to acute respiratory infection, can cause cardiopulmonary impairment and can have long-term physical and mental health consequences [1]. The measures implemented to contain COVID-19 impacted the health and wellbeing of people worldwide [2]. Besides the consequences of direct infection on individual health, the conjoint impact of the pandemic and the respective governmental policies regarding healthcare, social interaction, and the global economy also affected sexual and reproductive health (SRH) [3]. Indeed, the first research findings from the literature reviews demonstrate the COVID-19 pandemic’s broad influence on SRH [4,5,6,7]. Individuals faced unique challenges in their sexual lives and relationships, as well as in accessing SRH services during self-isolation and because of social distancing measures. The closing of healthcare facilities has led to disruptions in the supply of contraceptives and the provision of maternal and other SRH care services [8,9,10], possibly creating serious consequences in terms of contraception use, unplanned pregnancies, and sexually transmitted infections (STIs) [11,12].

In Germany, the very first case of COVID-19 was detected on 27 January 2020 [13]. Since then, Germany has seen four major waves of infection. In March 2020, a series of control measures, including self-isolation, quarantine, and mask-wearing, was adopted by the government [14]. These constituted major changes in the daily lives of the German population, which are mainly characterized by a significant reduction in social contact. COVID-19 has also challenged the German healthcare system and its professionals [15,16]. For example, surgical treatments that were not considered necessary were postponed in order to guarantee the capacity to care for COVID-19 patients. Even though the COSMO study, which investigates the attitudes and behaviours of Germans during the pandemic, found no deterioration in the provision of healthcare in Germany, its availability decreased in April 2020, as perceived by over 1000 German participants [15,17]. With regard to the actual healthcare utilization, various vaccinations, medical prescriptions, and consultations of outpatient care, such as those of general or specialized practitioners, declined, especially at the beginning of the pandemic, from March to May 2020 [17,18]. This decline especially impacted the utilization of preventive medical care, which encompass SRH services, such as mammographic screenings [18]. Because sexual healthcare, including STI screenings, pregnancy care, and regular gynaecological/urological check-ups, is often generally based on prevention, and is largely provided by general practitioners in Germany, the pandemic might, altogether, have a significant impact on SRH services.

The initial studies in Germany suggest that the pandemic and the protective measures affected mental health and the self-rated general health [19]. Looking at the pandemic impacts on sexual health in Germany, Schröder and colleagues used a qualitative methodology to describe the changes in the sexual experiences and desires of almost 250 German adults [20]. For instance, the participants reported an increased desire for physical closeness and intimacy, increased sexual fantasies, as well as an increased desire for greater emotional closeness in partnerships. Only a few international studies have explicitly investigated the underlying reasons for these changes. Panzeri and colleagues qualitatively identified the macrocategories of the causes for the increases and decreases in sexual activity, desire, arousal, and orgasms in the self-reports of Italian couples living together during the national lockdown [21]. The increases were related to more free time alone, but also to being together with partners, less stress, and more boredom. The decreases in sexual activity, desire, arousal, and orgasms, however, were related to increased stress and anxiety, forced co-living, and a lack of privacy, as well as to routine. While these results directly connect the pandemic and psychological conditions to increases or decreases in certain sexual behaviours, the identified macrocategories of the changes encompass numerous different aspects, of which some, e.g., stress, could cause either an increase or a decrease in the sexual activities of some individuals, as prior research has shown [22,23]. Similarly, Ballester-Arnal and colleagues identified the predictors of the perceived quality of the sex lives of Spanish participants, finding that, e.g., being female, or living with a partner, were associated with improvements, while high levels of stress and experiencing pandemic restrictions as “unbearable” were associated with a significant deterioration [24]. While the participants’ predictive traits are very specific, the perceived quality of one’s sex life is only one aspect, which encompasses many sexual behaviours and relevant cognitions, hence obscuring changes in the latter.

National online surveys show diverse changes in people’s sexual behaviours because of the COVID-19 pandemic and the respective containment measures across the globe. While social distancing measures led some to abstain from sexual activities [25], virtual sex, and the use of online dating, e.g., to find partners, connect to others, or to engage in sexual activity, increased in some studies and countries [25,26]. The accumulating evidence, e.g., [27], demonstrates an increase in the use of online pornography and masturbation [24,26,28]. Some studies further describe the gender differences in sexual activity, arousal, and desire [4,21,24], indicating that female sexuality might be affected to a greater extent by the pandemic situation, for instance, through increased sexual desire and activity, compared to men [4,24]. Moreover, in the aftermath of catastrophic events or natural disasters, which include the COVID-19 pandemic, intimate partner violence tends to accelerate [29,30,31]. An increase in online inquiries and calls to violence support institutions, of up to five times, was registered in Europe during 2020 [32]. However, Viero and colleagues stress the lack of quantitative studies assessing sexual and gender-based violence during the COVID-19 pandemic [7].

Studies on sexual behaviour and SRH in relation to COVID-19 are heterogeneous in design and are highly contextualized because of the differing pandemic and social conditions and governmental regulations. While a few online studies attempt to map the changes in the sexual behaviours of people during the pandemic a posteriori, at present, only Panzeri and colleagues have inquired about the specific reasons behind those changes [21]. However, this study only includes participants living with partners during the lockdown. Hence, the underlying psychological mechanisms provoking the behavioural changes associated with COVID-19 in the broader population remain unclear. Furthermore, in terms of the pandemic influences on sexual and gender-based violence, only limited secondary data are available at this point [7]. The current knowledge about COVID-19’s broader influence on SRH and sexual and gender-based violence worldwide rather resembles a patchwork of findings, which highlights the importance of global comparative research approaches to gaining insights into the social, structural and macrogeographical, as well as the unique national mechanisms, influencing the sexual lives and health of people during the pandemic.

Online studies have provided researchers with an opportunity to assess the pandemic effects on SRH in spite of the national lockdowns and the contract restrictions hindering population-based behavioural research [33]. In addition, survey data of large and diverse populations can be easily and feasibly collected by means of an online inquiry. Using this unique opportunity to create consensus for SRH measurement, the *International Sexual Health and Reproductive Health Survey in the time of COVID-19* (I-SHARE) provides a globally standardized SRH survey instrument [33]. I-SHARE, as an open science project, is paving the way for a coordinated and valid assessment of the COVID-19 outcomes on SRH worldwide, using multicountry comparative approaches. For the purpose of this study, the data collected in Germany are presented.

The main goal of this study is to describe the effects of the COVID-19 restrictions on partnerships and family relationships, sexual behaviour, access to contraceptives and maternal health care, abortion, and sexual and intimate partner violence as aspects of the SRH of adults in Germany. Furthermore, we aimed to investigate the changes in sexual behaviours, such as pornography consumption and masturbation, sexual satisfaction, and sexual problems. In addition to the quantitative methods, we also applied a qualitative approach to the open responses of the participants in order to identify the discrete categories of the reasons producing the changes in sexual behaviours, hence adding to and closing the gaps in the recent national and international findings on how the pandemic influences sexual experiences and behaviours.

## 2. Materials and Methods

### 2.1. Study Design

We conducted a cross-sectional online survey with adult participants using the internationally standardized I-SHARE protocol [33]. We adapted the globally standardised I-SHARE online survey instrument by providing a version in the German language, and by further adjusting the survey to suit the regional cultural and social conditions (see [33] for original English version). Our German I-SHARE survey comprises a maximum of 144 items, depending on the response behaviours and individual characteristics of the participants. Most of the items were single-choice formatted; however, seven items allowed for multiple choices, and one item was open for free responses. Depending on the participants’ demographics and response behaviours, the survey took 15–20 min to complete.

### 2.2. Study Participants and Recruitment

All individuals older than 18 years and living in Germany who provided their online informed consent were eligible for the study. The online link to the study was accessible via smartphones, tablets, and computers. The link was disseminated through email listservs, social media links, and the research team’s existing networks. Participation in the study was voluntary, anonymous, and uncompensated. The participants provided their consent online on the first page of the survey, after being informed about the aim and nature of the study. Hence, the participants were aware that the survey covers sensitive topics, such as sexual behaviour and experiences of sexual violence, before participation. In the introduction part and at the end of the survey, we provide the relevant contacts for further study inquiries, helplines (e.g., for gender-based and sexual violence) and services contacts, and data protection statements. Since the survey addressed sensitive topics, the participants could skip questions they did not wish to answer, or they could withdraw their participation and consent at any time. In this case, their data were not saved. The study was approved by the Local Psychological Ethics Committee of the University Medical-Centre, Hamburg (reference number: LPEK-0110), and was conducted in compliance with the Declaration of Helsinki [34].

The data collection started on 1 October 2020 and was finished by the end of December 2020. During this time, the German government implemented a wide variety of regulations to mitigate the spread of the virus, including an obligatory two-week quarantine for suspected cases of COVID-19, incidence-dependant restrictions of private meetings and travel, the recommendation to work from home, if possible, and limited school operation for selected classes only, while universities remained closed [14]. A further shutdown of all non-system-relevant retail stores and gastronomic services was enforced in November 2020. The restrictions that were imposed to control the COVID-19 pandemic also represented significant changes in the daily lives of the German population, which were mainly characterized by a shift towards domestic activities, and a critical reduction in the opportunities for social contact.

### 2.3. Measures

Besides sociodemographic information, the online survey included the following sections: (1) Compliance with COVID-19 social distancing measures; (2) Couple and family relationships; (3) Sexual behaviour; (4) Contraceptives; (5) Pregnancy and maternal health care; (6) Abortion; and (7) Sexual and intimate partner violence (see [33] for ISHARE study protocol and list of items per section). Table 1 depicts the selected sample items from each section. In several items, the participants were asked to report their respective behaviours and their frequencies, both before and during the COVID-19 social distancing measures.

### 2.4. Data Analysis

All of the data analysis was conducted using SPSS, Version 25 (Armonk, NY, USA). Single- and multiple-choice items were used for the quantitative analysis of the survey. We further used a qualitative coding approach for the open-response item [35]. All of the available cases with valid data were analysed.

### 2.5. Quantitative Analysis

In order to determine the changes in sexual behaviours and SRH due to the COVID-19 social distancing measures, the items from Sections 2–7 were descriptively analysed. All of the items in the “maternal health” section, as well as most of the items relating to abortion, were excluded from further analysis because the response rates were too low (i.e., ≤2). Two of the items of the “sexual behaviours” section were subjected to further statistical tests. For the item, “sexual problems in partnerships”, we conducted paired *t*-tests to determine the significant changes within the participants before and during the COVID-19 social distancing measures. Cohen’s *d*_z_ was used as a measure of the effect size for the paired *t*-tests, with │*d*_z_│ = 0.8 for large-sized effects, │*d*_z_│ = 0.5 for middle-sized effects, and │*d*_z_│ = 0.2 for small-sized effects [36]. For the second item, “sexual satisfaction”, we conducted a mixed two-factor ANOVA, with repetition on the factor, “sexual satisfaction”, in order to compare the differences between the reports of the participants in relationships, and the reports of the single participants, in terms of their sexual satisfaction, before and during the COVID-19 contact restrictions.

### 2.6. Qualitative Analysis

The open responses to the item, “*If some of your sexual behaviours have changed due to COVID-19 social distancing measures why do you think this happened*?”, from Section 3, were qualitatively analysed on the basis of the qualitative content analysis approach proposed by Mayring [35]. From the 326 obtained responses to this item, the preliminary content categories were identified inductively. This initial categorization was subsequently discussed among the research team, the scheme was adapted accordingly, and the responses were coded test-wise, using the adapted categorization. Afterwards, any learnings and unclarities were discussed, and the scheme was altered again. After several rounds of this iterative process, one researcher categorized the responses using the final categorization developed. The single responses containing several aspects of the multiple-content categories were coded multiple times.

## 3. Results

### 3.1. Sociodemographic Characteristics

We obtained valid responses from 611 adult participants. The sociodemographic characteristics are presented in Table 2. On average, the participants were 28 years old (*SD* = 8.3, *Med* = 26, *Min* = 18, and *Max* = 80). The majority of the participants were female, heterosexual, in relationships, without children, and lived in urban areas. Most had fully completed secondary school (90.7%, *n* = 554). The participants mostly reported no religion.

### 3.2. COVID-19 Impact on Daily Life

A total of 558 (91.4%) of the participants indicated that they complied with the COVID-19 social distancing measures “largely” or “strictly”. About one-quarter (28.8%, *n* = 175) of the participants had been self-isolating because of the risk of transmitting COVID-19. Seventeen participants (2.8%) had tested positive for COVID-19 at least once. The pandemic, and its impact on the German economy, produced changes in employment for 72.8% of our participants. Most of them indicated that they were still employed and paid, but that they were either working (partly) from home, on reduced time, or were unable to attend work (42.3%, *n* = 255). Some of the participants changed jobs (8.0%, *n* = 48) or lost their jobs or businesses (6.0%, *n* = 36). As a result, 26 participants (4.3%) stated that they were temporarily unemployed. Consequently, more than one-quarter of the participants claimed to have experienced a deterioration in their economic household situation (26.2%, *n* = 160), while a similar amount reported a partial (24.9%, *n* = 152), or a total (4.3%, *n* = 26), loss of income. The family, or household, compositions remained unchanged for most of our sample (88.2%, *n* = 539). At the time of the survey, more than half of the participants were living in partnerships. The majority of them lived in the same place with their partner for the whole period of the lockdown (46.2%, *n* = 177), or for part of the time (20.6%, *n* = 79). Generally, most of the participants in partnerships reported no changes in the emotional support provided by their partners during COVID-19 (60.6%, *n* = 189), with a tendency towards increased support (26.3%, *n* = 82), rather than decreased support (13.1%, *n* = 41). Of all the participants in partnerships, 44.7% (*n* = 139) reported an increase in relationship tension, e.g., because of conflicts. A total of 70 participants (18.3%) had recently separated from their partners, with more than half of them (52.9%, *n* = 37) reporting that the relationship ended during social distancing. For 10.1% (*n* = 7) of the separated participants, the social distancing measures had been the reason for their breakup. The majority of them remained single (84.3%, *n* = 59), while 15.7% (*n* = 11) subsequently had a new partner during social distancing.

### 3.3. Sexual Behaviours and Experiences

Of all the 611 participants, 98.7% (*n* = 603) stated that they had already had a sexual experience previously. In our sample, two participants (0.4%) reported that they were (probably) pregnant. Six participants (1.2%) recently delivered, while four participants (0.9%) had needed an abortion during the COVID-19 social distancing measures. A minority of participants reported using contraceptive methods irregularly (7.4%, *n* = 36) or not at all (10.5%, *n* = 48). A total of eight participants (1.9%) reported restricted access to contraception due to the social distancing measures.

Table 3 presents the relative frequencies of the participants’ reports of sexual satisfaction and sexual problems, prior to and during the COVID-19 restrictions. A paired *t*-test revealed that the participants’ reports of sexual problems in their partnerships (*t*(321) = −4.96, *p* < 0.001, *d*_z_ = 0.28), intraindividually, differed significantly, when comparing their reports before and during the COVID-19 social distancing measures with the small-sized effects [36].

**Figure 1:**
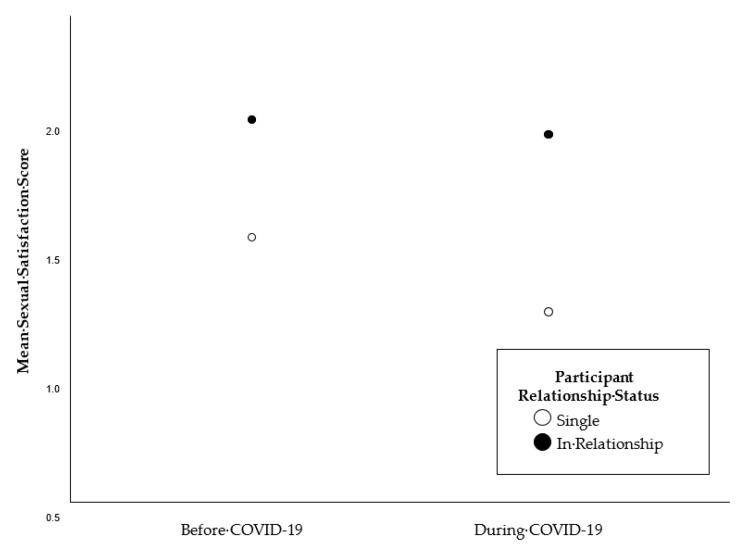
Mean sexual satisfaction scores before and during COVID-19 social distancing measures, by participant relationship status.

Table 4 depicts the relative frequencies of the reports of sexual satisfaction before and during COVID-19 for single participants, and for participants in relationships. Figure 1 illustrates the respective means. The mixed ANOVA yielded significant main effects for time (i.e., before and during the COVID-19 contact restrictions (*F*(1, 591) = 6.2, *p* = 0.01) and for the relationship status (*F*(1, 237) = 54.6, *p* < 0.001), and a nonsignificant interaction (*F*(1, N) = 2.5, *p* = 0.11).

Table 5 shows the reported changes in individual sexual behaviours. A total of 90.9% of participants (*n* = 289) in steady partnerships reported no changes in condom use during the COVID-19 social distancing measures. Changes in this regard were mainly reported by women (increased: 5.9%, *n* = 15; decreased: 5.1%, *n* = 13), with only one man (2.6%) also reporting a decrease in condom use with his partner. Of all the participants who were in a relationship before the COVID-19 social distancing measures, 117 (19.1%) reported at least one lifetime experience of sexual and gender-based violence, and most of them were females (86.3%, *n* = 101). A total of 73 participants, of which 82.2% (*n* = 60) were female, stated that they had experienced sexual violence during the COVID-19 social distancing measures.

### 3.4. Reasons for Changes in Sexual Behaviour

More than half of all of the participants (*n* = 326) replied to the open-response item, “*If some of your sexual behaviours have changed due to COVID-19 social distancing measures why do you think this happened?*”. Of all the responses, *n* = 55 were excluded since they were unrelated to COVID-19 or the subsequent containment measures. Furthermore, *n* = 105 of the participants’ responses contained several reasons, and were thus coded multiple times, finally resulting in *n* = 325 valid responses from *n* = 272 participants. A majority of these participants identified as females (81.6%, *n* = 222). A qualitative content analysis of the valid responses yielded 12 distinct categories of the reasons behind the participants’ reported changes in their sexual behaviours (see Table 6).

About one-quarter of the participants reported that changes in their sexual behaviours were caused by psychological problems, such as stress, loneliness, or boredom, as well as depression and anxiety symptoms due to COVID-19 and the social contact restrictions (“*I felt stressed and worried because of the circumstances*”; “*I felt panicky, stressed, and lonely*”). Furthermore, a substantial number of the participants often stated that the involuntary critical reduction in close contacts due to the COVID-19 social distancing measures, e.g., because of the cancellations of social events, restricted travel, or closed facilities, resulted in fewer possibilities to meet new partners, subsequently causing changes in their sexual behaviours (“*It is difficult to go on dates and meet new people*”). In this respect, *n* = 6 of these participants (1.8%) reported using online services to get to know or meet people virtually (“*I had less physical, but more virtual contacts*”). Following on from this, a considerable number of the participants stated that the changes in their sexual behaviours occurred because of the omission of other activities or distractions, which, hence, enabled them to spend more time by themselves or with their partner(s) (“*I spent more relaxed time at home with partner”*).

Apart from these individual reasons, COVID-19 also affected sexual behaviours because it caused tension in partnerships. The participants reported conflicts and relationships ending, too much physical and temporal closeness, and becoming used to, or bored of, one another at home (“*The constant being together has stressed me out”*). Furthermore, the physical separation between (casual) partners because of the pandemic restrictions caused changes in the participants’ sexual lives (“*I am in a long-distance relationship and my partner, and I could not meet during confinement”,* or *“I have retreated to monogamy”*). As illustrated, the social distancing measures against COVID-19 mainly comprised a shift in life towards the home for the German population. This shift also affected the participants’ sexual behaviours, e.g., because of the constant presence of others, because of spending work and leisure time at home, or because of a lack of input from the outside world (*“Since I am now working from home, we have more sex during the day, for example during lunch break”*). The pandemic changed the participants’ behaviours towards being cautious and avoiding meetings and other close physical contacts, e.g., public transport, either because of a fear of infection, compulsory self-isolation, or because of solidarity and the consideration of more vulnerable groups (*“Sex with changing partners during contact restrictions would not have been compatible with my good conscience”*).

In terms of the other more emotional reasons for the changes in their sexual behaviours, some participants indicated an increased need for physical closeness, intimate relationships, or partnership because of the COVID-19 social distancing measures (*“My need for closeness and comfort was much more intense”*). Similarly, some participants reported having made conscious decisions to change their sexual behaviours during the time of COVID-19, e.g., by working on their relationships and sex lives in their partnerships, as well as by focusing on their own sexuality, and sexual, physical, and mental health (*“We talked more about my sexual wishes and hence tried out new practices*”, or *“My attitude towards pornography has changed”*). Sexual desire also changed for a few participants. Of those who indicated changes, sexual desire increased for three of them, and decreased for seven of them (*“I felt less desire, less attractive, less motivated”*).

Moreover, two participants reported changes in their sexual behaviours because of decreases in their partners’ sexual desire (*“My partner has lost her sexual drive”*). Three participants reported that their motives for sexual activities changed because of COVID-19 and the subsequent containment measures, e.g., using sex to relax, to boost happiness, or to alleviate tension with a partner in a relationship (*“My partner and I had more sex because we got into arguments more easily during the time of COVID-19 and then had sex to reconcile”*).

## 4. Discussion

To the best of our knowledge, this study comprises the first online survey in Germany that quantifies the impact of the COVID-19 pandemic on the sexual and reproductive health of adults, in conjunction with a qualitative approach to uncover the respective reasons for the changes. Altogether, the current findings demonstrate that COVID-19 had an influence on the participants’ daily private and professional lives. Almost half of all of the participants living in partnerships reported increased tension between themselves and their partners during the pandemic. More than one-tenth of the participants, mostly females, stated that they had experienced sexual violence within partnerships during the COVID-19 social distancing measures. Across all genders, our participants further reported several changes in their sexual behaviours and experiences, including an overall decline in sexual satisfaction, increased sexual problems, and a tendency towards increased pornography consumption and masturbation. The main reasons for the changes in their sexual behaviours, reported subjectively by the participants, were psychological stress, followed by decreased social contacts, and increased time spent alone or with one’s partner. No significant changes in access to SRH services and commodities were registered. Only a small number of participants reported restricted access to contraception because of the social distancing measures.

In our sample, over 80% of the participants recalling sexually violent acts were female. This finding confirms the warnings of health professionals, researchers, and organizations that women might be particularly endangered by sexual and domestic violence during the pandemic [7,38,39]. As a natural disaster, the pandemic might directly contribute to an increase in sexual violence by creating conditions of stress and uncertainty [7], as well as more opportunities for domestic violence to occur because of confinement. Our findings on sexual violence within partnerships compare in magnitude with a study from Spain, where 2.8% of the participants felt sexually forced by another person during lockdown [24]. Hence, our results could map sexual violence during the COVID-19 pandemic, aligning with other systemic evidence, such as an increase in reports of intimate partner violence to the German police [40]. To solidify the information and quantify the increases in the occurrences of sexual and intimate partner violence, further specifically designed surveys and studies, combining data from several, especially institutional, sources, are needed. Most importantly, these results underline the necessity for broadly advertising and providing a comprehensive support system, offering specialized care and consultation, for those affected during critical times, such as during a pandemic.

In addition, the delineated increase in sexual problems, especially among female participants, aligns with results from Italy, where more women than men stated that they experienced difficulties in reaching orgasm during the pandemic [21]. Previous research has shown the heightened vulnerability of women to stress and depressive symptoms [41]. As a novel state of global emergency, the COVID-19 pandemic can be seen as a chronic stressor, highlighting the importance of building up adequate and accessible support systems for vulnerable groups.

Furthermore, the current findings reveal a decline in sexual satisfaction during the COVID-19 contact restrictions, besides the basic difference between the sexual satisfaction of the single and partnered participants. Even though a descriptive tendency is observable in our data, sexual satisfaction did not decline significantly for individuals not in partnerships. Furthermore, the descriptive decline in sexual satisfaction affected all genders, but it was especially pronounced in women and diverse individuals. Similarly, 43.5% of the participants reported the lower quality of their sex lives during the pandemic in Lehmiller and colleagues’ international online survey of over 1500 adults, mainly from the United States [28]. However, the first evidence from European studies suggests varying patterns concerning sexual satisfaction. For instance, in the online survey from Panzeri and colleagues, Italian couples reported no changes in sexual satisfaction during COVID-19 [21]. Yet, as in our study, sexual satisfaction varied more often for female participants. Likewise, more women reported a perceived general improvement in their sex lives and increases in desire compared to men in a Spanish study [24]. Altogether, the first results on the COVID-19-related changes in sexual satisfaction and desire, specifically, vary in magnitude and direction within the European countries. The structure of our obtained data does not always allow for the valid inferential statistical testing of the descriptive differences between the genders. Hence, further research efforts are necessary to precisely assess these international variations in the future in order to uncover the differing mechanisms, and how they affect sexual desire and satisfaction, while incorporating the factor of gender.

Interestingly, most of the participants in our sample did not report significant changes in their sexual behaviours. However, some tendencies towards changes were seen. First, masturbation and pornography consumption tended to increase across genders, with more pronounced increases in men. Findings of other national studies also demonstrate self-reported increases in solo-sex activities, especially in men [24,26]. Theoretical frameworks, e.g., [41], and study results, e.g., [42,43,44], further support the notion of sex used as a coping strategy to alleviate negative emotional states or stress. Psychological stress has been the strongest self-reported reason for the changes in the participant’s sexual behaviours in the current study. Likewise, a small proportion of participants in Schröder and colleagues’ qualitative study reported increases in, along with changes in the meaning of, masturbation [20]. Comparable to the reasons provided for the changes in the sexual behaviours in our study, the participants stated that they masturbated to distract themselves or to alleviate boredom. Hence, the self-reported increases in pornography consumption and masturbation are not surprising. At the same time, sexual activity with steady or casual partners tended to decrease in our sample, aligning with some other studies from Australia, the United Kingdom, and Italy [26,45,46]. Coombe and colleagues point out decreases in sexual activity with casual partners and girl- or boyfriends, while the frequency of sexual activity between spouses increased [26]. Further supporting this notion, the results from previous research indicate a decrease in sexual activity outside partnerships, and an increase in sexual frequency in couples living together during lockdown [20,21]. Considering these distinctions, the decrease noted in our sample could be explained by the confinement and contact restrictions, which posed an obstacle to meeting sexual partners for people not living together with a partner, which most of the participants in our sample did. A limiting effect of the COVID-19 social distancing measures on the availability of sexual partners also coincides with the participants’ self-reported reasons for the changes in their sexual behaviours, with fewer options for social contact being named as the second most frequent reason. This could also help to explain the observed increase in solo-sex activities. Similar to the results concerning sexual satisfaction, some studies remark on the greater variability in the sexual activities of women [12,21]. This tendency can be found in our sample as well, with women reporting greater increases and decreases in sexual activities compared to men. Additionally, the female participants in our study reported increases in acts of physical closeness, such as hugging, kissing, or holding hands.

Our inductive qualitative approach provides further insights into the participants’ self-suspected reasons underlying the reported changes in their sexual behaviours. Supporting widespread social narratives, the pandemic and social contact restrictions, first and foremost, constituted a major strain on the psychological wellbeing of the participants, which strongly affected their sexual lives. In our study, the psychological aspect was closely tied to the omission of social contacts. Often, the participants directly linked their feelings of psychological stress to the limited possibilities to see or meet new (casual) sexual partners, or other people in general. Three other studies from Europe have investigated the reasons for the changes, largely supporting and extending these results [20,21,24]. Although the categorizations differ because of the qualitative methodology, many concurring aspects of the reasons, besides the ones already mentioned, can be found; for example, an increased need for physical closeness and partnerships, shifts towards monogamy, more intense dealings with sexuality and partnerships, restrained dating because of the consideration for groups more vulnerable to COVID-19, and the impediment to meeting new partners because of the contact restrictions. Stress, anxiety, and depressive symptoms negatively influenced aspects of the participant’s sexual lives, such as desire and satisfaction, in line with previous research, e.g., [47,48,49]. In other studies, psychological stress has, on the one hand, led to a decrease in sexual activity in some participants, while, on the other hand, it has led to an increase in others, who use it as a coping mechanism to counterbalance negative emotions, resulting in increased sexual activity [12,21]. Furthermore, the omission of social opportunities equipped the participants with more free time in their everyday lives. Most participants, who stated this as the main reason for the changes in their sexual behaviours, found the time gained to be positive, and stated that it allowed them to invest more time in themselves or their partnerships, and to enjoy the closeness and the relaxing deceleration of life. This aligns with the results from reports of German adults [20] and Italian couples [21], who named the increased time and the closeness between partners as the factors contributing to increases in sexual desire, arousal, and the frequency of sexual activity and orgasms. However, considering that many participants remarked on feelings of loneliness, anxiety, worry, insecurity and boredom, this positive evaluation seems, rather, to be an exception. Furthermore, many of the participants reported the stressful aspects of increased closeness and time spent at home during the pandemic, such as a lack of privacy, being overloaded by one’s partner, or even enhanced conflict in partnerships, confirming the results from Spain and Italy [21,24].

Generally, studies, e.g., [50], show that women tend to participate more in surveys. Furthermore, our recruitment strategy was mostly conducted online via social media platforms, such as Facebook and Instagram. The convenience of this recruitment strategy is a possible explanation for the gender imbalance towards females and those of a young age, and, hence, the childlessness of our participants. Thus, our findings are not generalizable to the German population. Our results can only be seen as preliminary, and they encourage future research to find a more nuanced perspective, especially on the predictors of the changes in sexual desire and the frequency of sexual activity during situations of stress throughout society. Moreover, although online surveys provide an opportunity to collect and analyse data quickly during lockdown conditions, it can lead to the exclusion of participants who do not have access to the Internet, e.g., those from rural regions and with lower socioeconomic statuses. Further taking into consideration the heterogeneity of the national surveys’ quantitative findings, the mixed methods should be given a stronger focus in order to gain and investigate the relevant variables in the sexual behaviours and experiences of individuals during the COVID-19 pandemic.

## 5. Conclusions

This online survey demonstrated changes in the sexual health behaviours of adults living in Germany during the COVID-19 pandemic, and identified important challenges that should be addressed, such as gender-based and intimate partner violence. In order to support sexual health in the context of the social distancing measures during the COVID-19 pandemic, e-Health programs could be useful to individuals for developing short-term coping strategies, alleviating stress, and possibly preventing sexual problems. These programs should, for example, promote communication about sexual problems, as well as discrepancies in the sexual desire of partners in relationships.

## Figures and Tables

**Table 1:** Sample items.

Section	Sample Items
Compliance with COVID-19 measures	How much have you complied with the contact restrictions so far? Have you been in domestic quarantine in the past, for example, because you had symptoms of COVID-19, or because you had contact with an infected person, or because you had returned from a severely affected country?
Couple and family relationships	Has your family situation changed during the contact restrictions? During the COVID-19 contact restrictions, how often did you experience tension in your relationship with your partner?
Sexual behaviour	During the COVID-19 contact restrictions, how satisfied were you with your sex life? During the COVID-19 contact restrictions, how often did you consume pornography?
Contraception	Are you or your partner currently doing anything to prevent or delay pregnancy, including condom use, contraceptive methods, etc.? Did the contact restrictions make it difficult for you to access contraceptives?
Pregnancy and maternal healthcare	If you are currently pregnant, was your pregnancy planned? Did you miss or postpone a prenatal care appointment during the contact restrictions?
Abortion	Did you need an abortion (termination of pregnancy) during the contact restrictions? Has the COVID-19 situation prevented you from seeking or having an abortion?
Sexual and intimate partner violence	During the COVID-19 contact restrictions, has your partner hit you, pushed you, kicked you, choked you, or thrown something at you that could have hurt you? During the COVID-19 contact restrictions, has your partner physically forced you to have sexual intercourse even though you did not want to?

**Table 2:** Sociodemographic characteristics of participants.

Sociodemographic Characteristic	*n* (%)
**Sex** (*N* = 611)	
Female	505 (82.7)
Male	105 (17.2)
Other	1 (0.2)
**Gender** (*N* = 611)	
Female	486 (79.5)
Male	98 (16.0)
Other	27 (4.4)
**Sexual orientation** (*N* = 611)	
Heterosexual	360 (59.0)
Homo-/bi-/pan-/asexual	206 (33.8)
Other	44 (7.2)
**Relationship Status** (*N* = 610)	
Single	246 (40.3)
In relationship	356 (58.3)
Other *	9 (1.5)
**Children** (*N* = 611)	
No	549 (89.9)
Yes (M = 1.82, SD = 1.2)	62 (10.2)
**Education** (*N* = 611)	
Low or none	15 (2.5)
Secondary	579 (94.8)
**Religion** (*N* = 611)	
No religion	397 (65.0)
Protestant/Catholic	198 (32.4)
Other	16 (2.6)
**Area of living** (*N* = 611)	
Urban area	486 (79.6)
Rural area	122 (20.0)
Other	3 (0.5)

* Note: Since the relationship status was assessed using a multiple response item, ambiguous responses that could not be assigned to either category, “Single” or “In relationship”, were classified as “Other”.

**Table 3:** Sexual satisfaction and sexual Problems, before and during COVID-19 social distancing measures, by participant gender.

Items and Choices	Before COVID-19 (%)			During COVID-19 (%)		
How satisfied were you with your sex life?	Female	Male	Diverse	Female	Male	Diverse
	(*n* = 481)	(*n* = 96)	(*n* = 25)	(*n* = 482)	(*n* = 95)	(*n* = 25)
Not at all satisfied	65.6	28.1	6.3	70.4	25.4	4.2
Not very satisfied	78.8	19.4	1.8	81.5	15.2	3.4
Somewhat satisfied	81.4	12.2.68	5.9	79.8	13.9	6.3
Very satisfied	81.7	15.7	2.6	84.3	14.8	0.9
How often have you or your partner experienced sexual problems?	Female	Male	Diverse	Female	Male	Diverse
	(*n* = 310)	(*n* = 56)	(*n* = 15)	(*n* = 258)	(*n* = 52)	(*n* = 12)
Never	33.9	37.5	26.7	30.6	42.3	33.3
Sometimes	56.8	50.0	73.3	54.7	40.4	58.3
Often	9.4	12.5	-	14.7	17.3	8.3

*Note:* The category “Diverse” refers to participants who identify as either both female and male, neither female nor male, or as “other”.

**Table 4:** Sexual satisfaction before and during COVID-19 social distancing measures, by participant relationship status.

Items and Choices	Before COVID-19 (%)		During COVID-19 (%)	
How satisfied were you with your sex life?	Single	Rel.	Single	Rel.
	(*n* = 238)	(*n* = 355)	(*n* = 238)	(*n* = 355)
Not at all satisfied (0)	81.3	18.6	80.3	19.7
Not very satisfied (1)	50.9	49.1	49.1	50.9
Somewhat satisfied (2)	36.6	63.4	32.2	67.8
Very satisfied (3)	21.4	78.6	17.1	82.9
Mean Score (SD)	1.53 (0.82)	1.99 (0.74)	1.24 (0.91)	1.93 (0.82)

*Note*: Rel. = In a relationship. The Shapiro–Wilk test assessed the non-normal distribution for all four groups (*p* < 0.05). However, all groups were sufficiently large (*n_single_* = 238, *n_relationship_* = 355, *n_before COVID_* = *n_during COVID_* = 592), and seem to be approximately normally distributed upon optical inspection. Furthermore, the ANOVA has proven to be robust to violations of the normal distribution [37]. There was homogeneity among the covariances, as assessed by Box’s test (*p* = 0.117). However, Levene’s test revealed the nonhomogeneity of the error variances (*p* < 0.01). Hence, we decided to conduct a robust ANOVA using the WRS2 package in R.

**Table 5:** Changes in sexual behaviours during COVID-19 social distancing measures.

Sexual Behaviours	%		
	Female	Male	Diverse
Sexual activities with steady partner	*n* = 258	*n* = 52	*n* = 12
Decreased	34.9	30.8	41.7
Stayed the same	46.5	53.8	58.3
Increased	18.6	15.4	-
Hugging, kissing, holding hands with partner	*n* = 257	*n* = 52	*n* = 12
Decreased	15.2	15.4	25.0
Stayed the same	48.6	67.3	58.3
Increased	36.2	17.3	16.7
Sexual activities with casual partner	*n* = 476	*n* = 95	*n* = 25
Decreased	17.2	26.3	36.0
Stayed the same	68.5	64.2	60.0
Increased	14.3	9.5	4.0
Condom use with casual partner	*n* = 158	*n* = 38	*n* = 7
Decreased	4.4	2.6	-
Stayed the same	93.0	94.7	85.7
Increased	2.5	2.6	14.3
Masturbation	*n* = 481	*n* = 95	*n* = 24
Decreased	24.9	10.5	8.3
Stayed the same	43.9	55.8	62.5
Increased	31.2	33.7	29.2
Sending/receiving (semi-) naked pictures/videos	*n* = 476	*n* = 95	*n* = 24
Decreased	11.3	6.3	8.3
Stayed the same	76.5	86.3	79.2
Increased	12.2	7.4	12.5
Pornography consumption	*n* = 477	*n* = 96	*n* = 24
Decreased	15.7	9.4	12.5
Stayed the same	67.3	58.3	70.8
Increased	17.0	32.3	16.7
Online sex	*n* = 473	*n* = 95	*n* = 25
Decreased	2.1	2.1	4.0
Stayed the same	94.7	93.7	92.0
Increased	3.2	4.2	4.0

*Note:* The category “Diverse” refers to participants who identify as either both female and male, neither female nor male, or as “other”.

**Table 6:** Categories of reasons for changes in sexual behaviours, and respective frequencies by gender.

Categories	*f (%)*		
	Female (*n* = 266)	Male (*n* = 43)	Diverse (*n* = 16)
Psychological stress	61 (83.6)	11 (15.1)	
Fewer options for social contact	49 (77.8)	9 (14.3)	5 (7.9)
More time	42 (85.7)	7 (14.3)	-
Tension in partnership	32 (94.1)	1 (2.9)	1 (2.9)
Separation from (casual) partner(s)	26 (86.7)	3 (10.0)	1 (3.3)
Shift to domestic life	22 (81.5)	4 (14.8)	1 (3.7)
Caution and fear of infection	8 (61.5)	4 (30.8)	1 (7.7)
Increased need for closeness	10 (83.3)	-	2 (16.7)
Reflecting sexual behaviour and mental health	7 (70.0)	1 (10.0)	2 (20.0)
Change in sexual desire	7 (70.0)	1 (10.0)	2 (20.0)
Change in motives for sexual activity	1 (50.0)	1 (50.0)	-
Change in partner’s sexual desire	1 (50.0)	1 (50.0)	-

*Note:f* = frequency of responses.

## Data Availability

Data available upon reasonable request.
